# The effect of antibiotics on the gut microbiome: a metagenomics analysis of microbial shift and gut antibiotic resistance in antibiotic treated mice

**DOI:** 10.1186/s12864-020-6665-2

**Published:** 2020-03-30

**Authors:** Lei Xu, Anil Surathu, Isaac Raplee, Ashok Chockalingam, Sharron Stewart, Lacey Walker, Leonard Sacks, Vikram Patel, Zhihua Li, Rodney Rouse

**Affiliations:** 1 U. S. Food and Drug Administration, Center for Drug Evaluation and Research, Office of Translational Science, Office of Clinical Pharmacology, Division of Applied Regulatory Science, HFD-910, White Oak Federal Research Center, 10903 New Hampshire Ave, Silver Spring, MD 20993 USA; 2 U. S. Food and Drug Administration, Center for Drug Evaluation and Research, Office of Medical Policy, White Oak Federal Research Center, 10903 New Hampshire Ave, Silver Spring, MD 20993 USA

**Keywords:** Gut microbiome, Next generation sequencing, Antibiotics, Antibiotic resistance, Metagenome

## Abstract

**Background:**

Emergence of antibiotic resistance is a global public health concern. The relationships between antibiotic use, the gut community composition, normal physiology and metabolism, and individual and public health are still being defined. Shifts in composition of bacteria, antibiotic resistance genes (ARGs) and mobile genetic elements (MGEs) after antibiotic treatment are not well-understood.

**Methods:**

This project used next-generation sequencing, custom-built metagenomics pipeline and differential abundance analysis to study the effect of antibiotic monotherapy on resistome and taxonomic composition in the gut of Balb/c mice infected with *E. coli* via transurethral catheterization to investigate the evolution and emergence of antibiotic resistance.

**Results:**

There is a longitudinal decrease of gut microbiota diversity after antibiotic treatment. Various ARGs are enriched within the gut microbiota despite an overall reduction of the diversity and total amount of bacteria after antibiotic treatment. Sometimes treatment with a specific class of antibiotics selected for ARGs that resist antibiotics of a completely different class (e.g. treatment of ciprofloxacin or fosfomycin selected for cepA that resists ampicillin). Relative abundance of some MGEs increased substantially after antibiotic treatment (e.g. transposases in the ciprofloxacin group).

**Conclusions:**

Antibiotic treatment caused a remarkable reduction in diversity of gut bacterial microbiota but enrichment of certain types of ARGs and MGEs. These results demonstrate an emergence of cross-resistance as well as a profound change in the gut resistome following oral treatment of antibiotics.

## Background

Currently, multiple health organizations including the U. S. Centers for Disease Control and Prevention [1], the World Health Organization [2] as well as others [3] have identified proliferation of antimicrobial resistance as a global crisis. Antibiotics are globally used in the treatment of bacterial infections [4–6] and typically kill most antibiotic-susceptible bacterial populations in a relatively short time. However, a small fraction of bacteria can survive and represent a major concern for emergent antibiotic resistance and recurrent infection [7]. Dependent upon mechanism of action, resistant bacteria may revert to a non-resistant state in the absence of antibiotics [8]. However, when novel genetic mutations or resistance conducting plasmids appear, antibiotic-resistant strains can persist in the absence of this selective pressure contributing to the reservoir of antibiotic resistance [9].

The gut microbiome has been increasingly implicated in disrupting health and behavior [10–14]. Recent molecular studies discovered that the taxonomic composition of human intestines is host specific [15, 16], relatively stable over a time [16, 17], and linked to many human diseases [18–22]. Microbial communities in the gut produce extensive amounts of metabolic products, interact intimately with human cells, and play an important role in maintaining many physiological processes and functions [23, 24]. These communities can be dramatically disturbed after the oral use of antibiotics and lead to profound alterations in the relevant abundance of different bacterial species, the rise of new species, and/or complete eradication of existing species [9, 25, 26]. While these are unintended off-target effects of antibiotic use, large shifts in community composition of bacteria linked to health and well-being [27] could have potential repercussions for the host, including overgrowth of antibiotic-resistant species. In addition, it is presently unclear how large changes in taxonomic composition might influence the spread and stabilization of antibiotic resistant genes in bacterial populations particularly with use of antibiotics [9]. The resistome may potentially change drug efficacy and safety through interactions that modulate drug metabolism [28–30]. One long-standing concern is that the use of single or multiple systemic antimicrobials may select for resistant mutants in the gut flora, creating the threat of new untreatable infections. Recently CDC launched Antibiotic Resistance (AR) Solutions Initiative to understand resistance and to explore new strategies and innovative approaches to slow antibiotic resistance [27]. The first step in this process is to better understand the shifts in community composition in response to antibiotic treatments in the context of treatment for infection.

The public platform of analysis, Quantitative Insights into Microbial Ecology (QIIME), and other 16S rRNA and 18 s rRNA sequence analyses are widely used for gut microbiome taxonomical composition analysis [31, 32]. Metagenome sequencing and analysis have been used extensively for studying microbial communities as well as for *bacterial* gene mutation and genome variation analyses [33]. MetaPhlAn is a public platform computational tool for profiling the composition of microbial communities (Bacteria, Archaea, Eukaryotes and Viruses) from metagenomic shotgun sequencing data at the species-level [34]. Metaxa2 is a software tool capable of extracting partial and full-length small subunit (16S/18S) rRNA and large subunit (23S/28S) sequences from metagenomic shotgun sequencing data and assign taxonomic classification to the extracted sequences by comparing them against publicly available reference databases [35]. In the present project, metagenome sequencing data derived from the gut of mice treated for urinary tract infection (UTI) were analyzed using MetaPhlAn [34] and Metaxa2 [35] to characterize community composition at different timepoints during antibiotic treatment. Changes in gut resistome were studied by mapping sequences against the Comprehensive Antibiotic Resistance Database (CARD) [36]. The UTI mouse model was created by instilling uropathogenic *E. coli* into the urinary bladder via transurethral catheterization. Beginning 24 h after bacterial inoculation, treatment was initiated with ampicillin (amp), ciprofloxacin (cipro), or fosfomycin (fosfo); each a commonly used antibiotic in clinical UTI treatment [37]. The UTI model was used as UTI is one of the most common bacterial infections encountered in clinical practice in Europe and North America and *E. coli* was used as the experimental organism because it is the most prevalent (75–95%) bacteria found in common clinical UTI [37].

The initial objectives of the work include tracking the evolution of resistance of the pathogens in the bladder and characterizing the similarities and differences in influence of antibiotics with differing mechanisms of action on the gut resistome and community composition. While work about the first objective was published elsewhere [38], this manuscript reports findings about the second objective and characterizes the changes in the gut microbiome. The initial endpoints of characterization were shifts in gut microbial community and changes in relative abundance of recognized antibiotic resistance genes, or identification of emergent antimicrobial-resistant genes.

## Results

### Antibiotic-induced changes in taxonomic composition of mouse gut

**Fig. 1:**
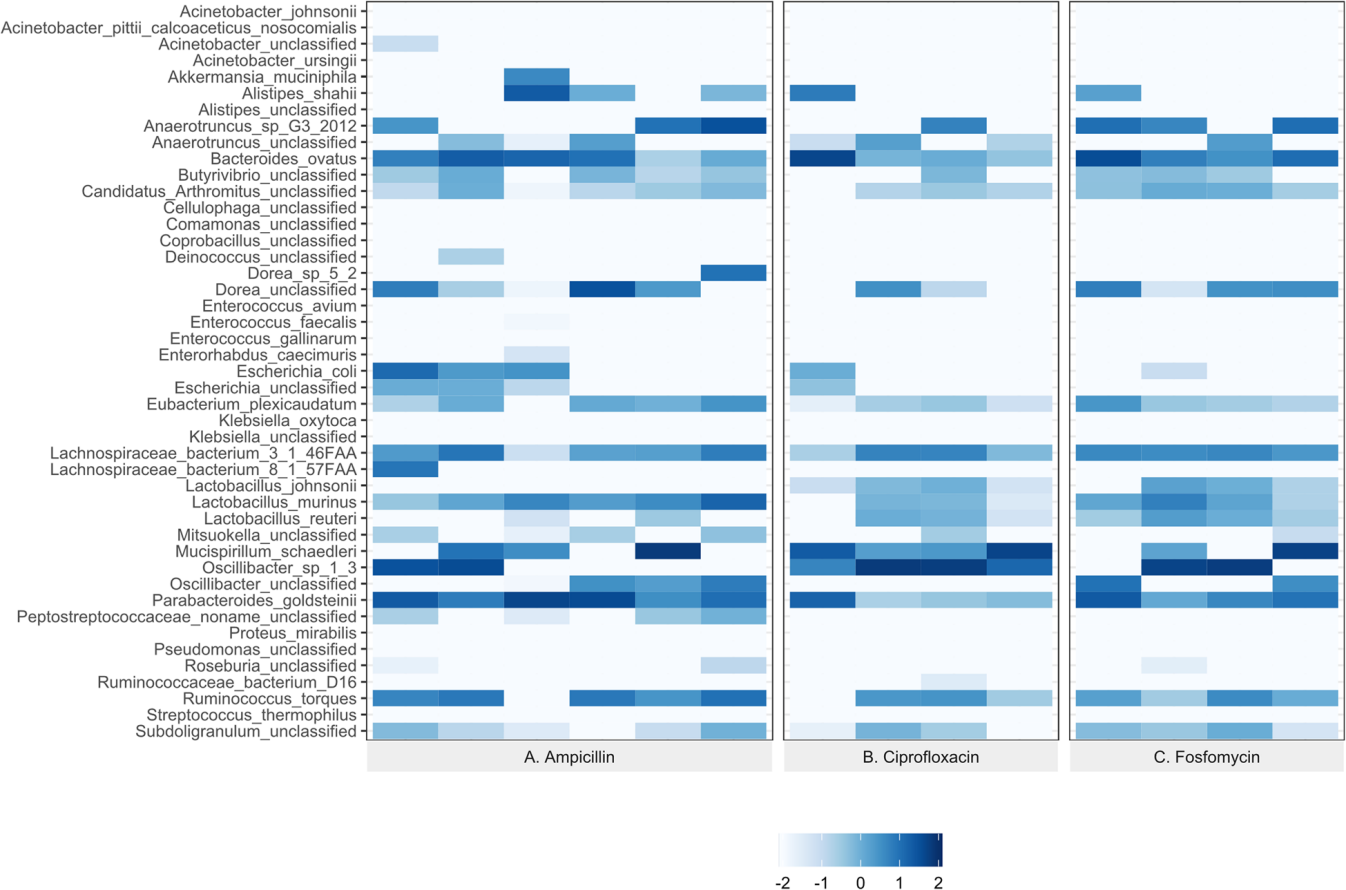
Control group of gut microbiome analysis. Heatmap representing log-transformed relative abundance of the bacterial species in each control group (**a**, **b**, **c**). A total of 36 individual bacteria species were identified from the three control groups

Figure 1a-c presents the control samples allowing a comparison of species relative abundance before treatment with each antibiotic. There was individual variability in the identified species, but each control group had a very similar species abundance pattern. A total of 36 bacterial species were identified from the gut microbiota of the three control groups of mice using the Metaphlan2 [34] reference genome (Supplementary Table 7).

**Fig. 2:**
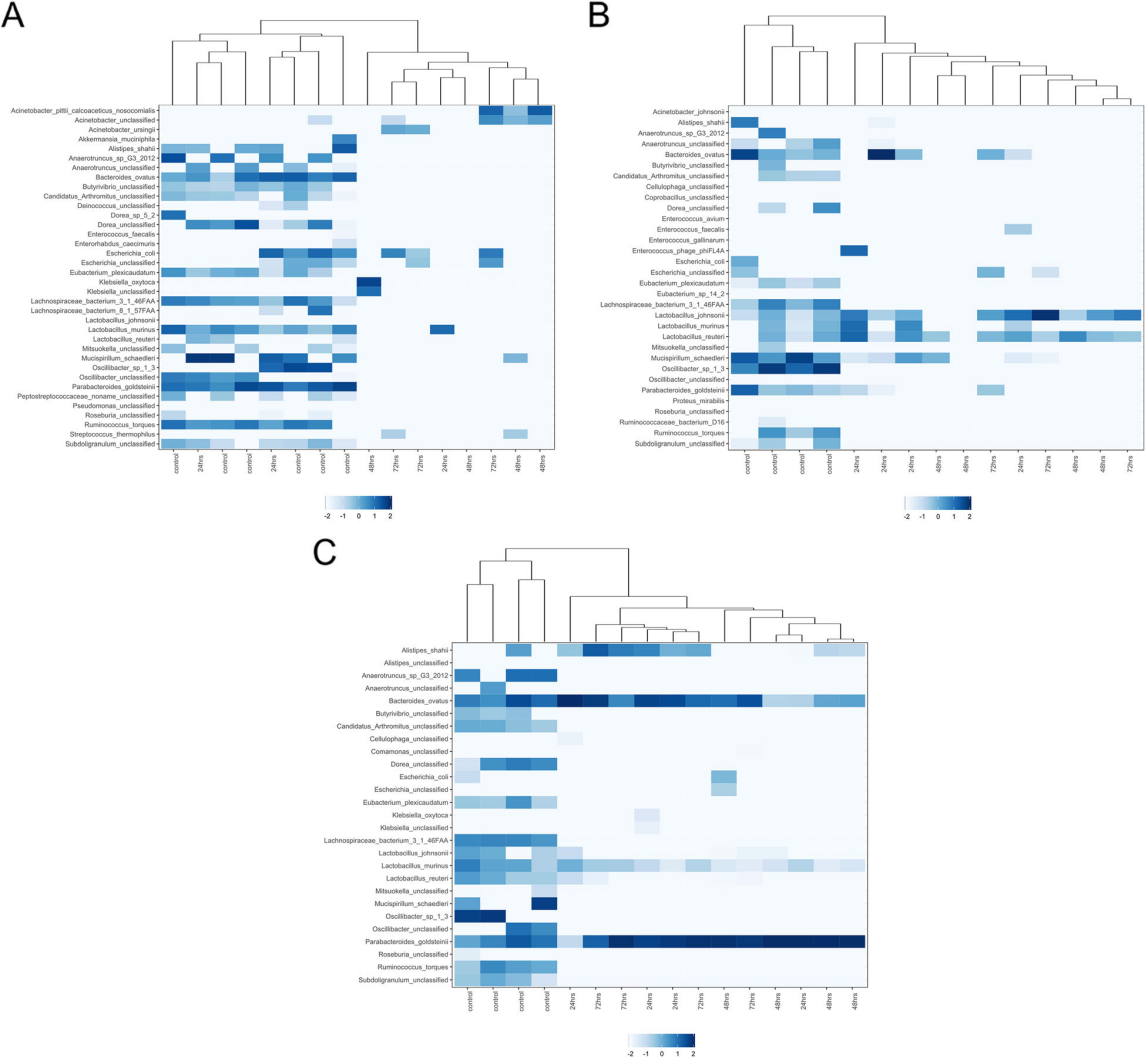
**a**-**c** Heatmap with dendrogram demonstrating log-transformed relative abundance and clustering of microbial species in the mouse gut. Relative abundance influenced by Ampicillin (**a**), Ciprofloxacin (**b**), or Fosfomycin (**c**) after 24, 48, and 72 h of treatment. Note the clustering together of control versus the clustering together of treated mice. Species were ordered in each graph to facilitate visualization of clustering. Color indicates the relative abundance data after log transformation

After treatment, each antibiotic produced increased relative abundance in different species but also shared a large common list of species that were eradicated or undetectable after treatment. Figure 2a-c shows that in each antibiotic exposure the microbiota of treated animals generally clustered together and were hierarchically separable from control animals that clustered together separately from the treated mice indicating that treated mice microbiotas were more similar to one another than to their respective controls with the exception of two treated mice in the post 24-h amp exposure group that clustered with the control group. This could be due to an inconsistency in delivery of the antibiotic dosage, variation in absorption by the individual mice or a variation in ampicillin sensitivity of gut community of individual mice. This general trend in sample clustering was verified with ordination plots (PCoA) generated using 16S rRNA and ARG abundance data, Figs. 6 and 8, respectively. Naïve (uninfected) and infected controls consistently clustered together across all the antibiotic studies. This is confirmed with a PCoA plot (Supplementary Fig. 1) based on Bray–Curtis dissimilarity of ARG abundances of all control and treatment samples from all three antibiotics.

**Fig. 3:**
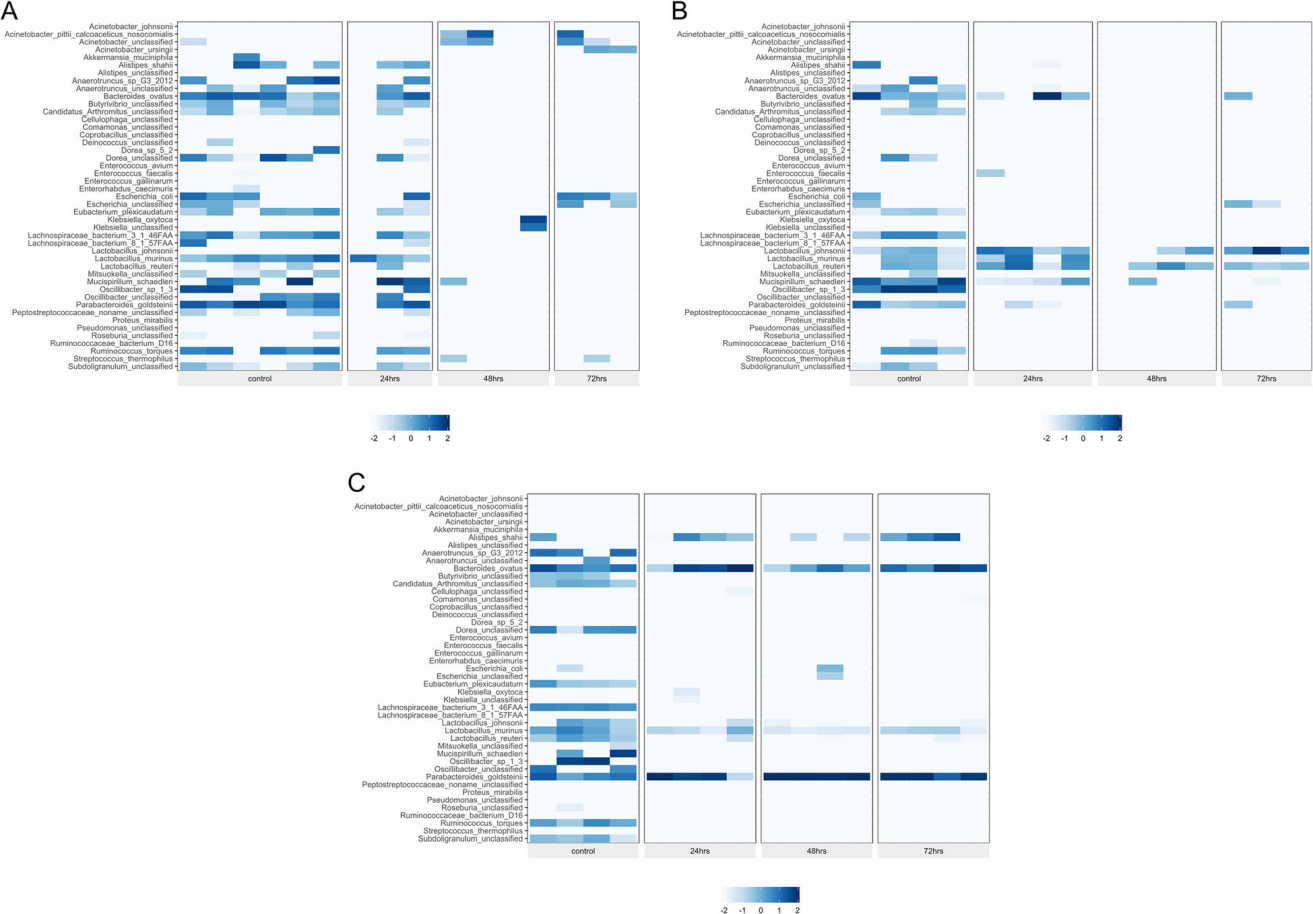
**a**-**c** Heatmap presentation of antibiotic modulation of the log-transformed relative abundance of microbial species in the gut by Ampicillin (**a**), Ciprofloxacin (**b**), or Fosfomycin (**c**) after 24, 48, and 72 h of treatment, respectively. These heatmaps represent the species listed in the same order across each heatmap to allow comparisons. Color indicates the relative abundance data after log transformation

In Fig. 3a-c, the change in species relative abundance caused by the antibiotic exposures can be visualized. With each antibiotic treatment, a large percentage of the bacteria identified pre-treatment were absent or greatly diminished after treatment. Observing the change in heatmap patterns, impacted species appear similar across the three antibiotics, although as with abundance of species in controls, there was some variability. Fosfo had an immediate and persistent influence on the number of species detected. By 24-h after a single treatment, all the change that was to take place had occurred and the remaining species became the prevalent species for the remainder of the experiment. This change in community composition is depicted in PCoA plot (Fig. 6c) as well. Box plots in Fig. 5c shows changes in Shannon Diversity where a similar pattern was observed for fosfo. With cipro treatment, major changes were also observed within 24 h, but it took 48 h for some of the bacterial species to be maximally impacted. The species that assumed prominence post-cipro were different from those that did so after fosfo treatment. Figures 5b and 6b confirm a similar trend for cipro. Treatment with amp resulted in more variation in the timing of effects. By 48 h post-treatment, all the influence of treatment had been seen in the species that were diminished and in those that rose to highest relative abundance. PCoA plot and box plots (Figs. 5a and 6a) show a gradual shift in the relative abundance and Shannon Diversity index of the community. Multiple Acinetobacter species became part of the enriched microbiota following amp treatment, but similar Acinetobacter population enrichment was not observed with either cipro or fosfo. The most prominent emergent species noted for fosfo were greatly diminished with amp and cipro treatment.

Twenty-four hours after treatment with amp, most species noted in controls were still detectable as shown in Supplementary Table 4, while after 48 to 72 h of treatment, most pre-treatment species (including *Eubacterium plexicaudatum, Lachnospiraceae bacterium 3 1 46FAA, Lachnospiraceae bacterium 8 1 57FAA, Oscillibacter sp. 1–3, Oscillibacter unclassified, Anaerotruncus sp. G3–2012, Anaerotruncus unclassified, Ruminococus torques, Butyrivibrio unclassified, and Enterococcus faecalis)* were undetectable except for *Mucispirillum schaedleri* and *Streptococcus thermophilus* that were detectable in limited samples. Interestingly, *Escherichia* species, such as *Escherichia coli* and *Escherichia unclassified* were still present after 72 h of treatment and multiple species of Acinetobacter, such as *Acinetobacter pittii calcoaceticus nosocomialis, Acinetobacter ursingii and Acinetobacter unclassified* arose to become the prominent species in the 48- and 72-h treatment groups (Figs. 2a and 3a). *Acinetobacter* genus is one of the genera that was found to have a statistically significant enrichment (based on 16S rRNA analysis) after treatment with Ampicillin (Table 1A).

**Table 1:**
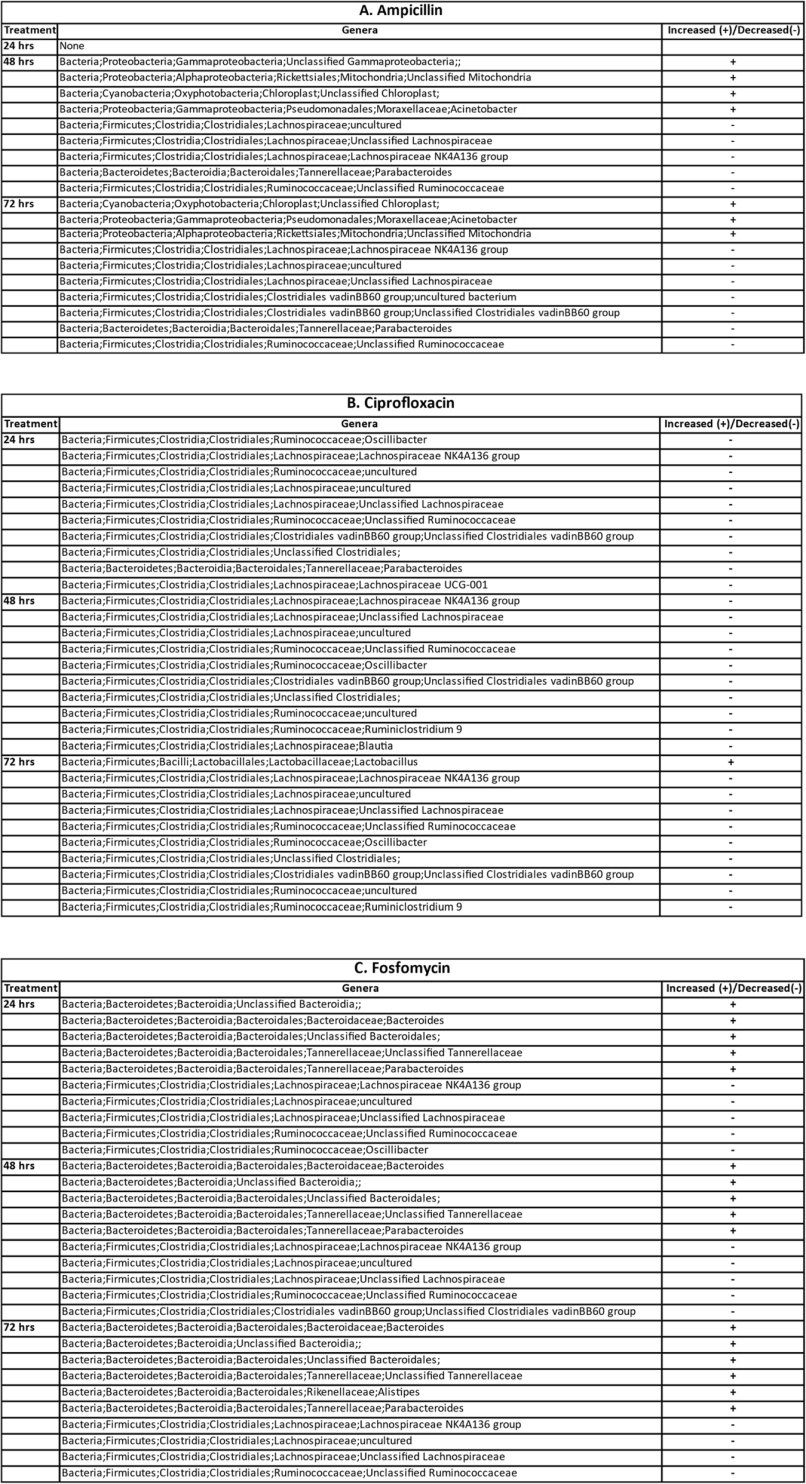
A-C Top 10 statistically significant changes in genera after oral treatment with Ampicillin (A), Ciprofloxacin (B), or Fosfomycin (C)

For each antibiotic cohort, the top 10 statistically significant changes in  bacterial genera determined using edgeR are listed in tabular form. Plus sign in the last column indicates that the genera count increased after treatment and a minus sign indicates a decrease in the count after treatment. A complete list of these genera for each cohort/treatment combination along with their log2 fold change, *p*-value & FDR values reported by edgeR are provided in Supplementary Table 2A-I

Cipro impacted species are captured in Supplementary Table 5. After 24 h treatment, unclassified species of *Anaerotruncus*, *Oscillibacter*, *Dorea* were minimally detected with species of *Eubacterium, Lachnospiraceae, Oscillibacter, Anaerotruncus,* and *Escherichia* undetectable after 48 h treatment. Though its abundance in the control group was not high, *E. coli* was not identified in the microbiota 24 h after treatment with cipro while *Lactobacillus johnsonii, Lactobacillus reuteri, Lactobacillus murinus* emerged as the dominant bacteria increasing in relative abundance (Figs. 2b and 3b). 16S rRNA analysis confirms this result in that the *Lactobacillus* genus showed a statistically significant increase in relative abundance (Table 1B).

The fosfo influence on species relative abundance is shown in Supplementary Table 6. *Pseudomonas unclassified, Mucispirillum schaedleri, Eubacterium plexicaudatum, Anaerotruncus sp. G3–2012 and Anaerotruncus unclassified, Oscillibacter sp. 1–3 and Oscillibacter unclassified,* the majority species of *Lactobacillus, such as Lactobacillus johnsonii* and *Lactobacillus reuteri* were reduced by over 90% with fosfo exposure, although *Lactobacillus murinus* was an exception experiencing minimal change. *E. coli* was not identified in the 24-h post-treatment group. With large groups of the bacterial population undetectable, two species, *Parabacteroides goldsteinii* and *Bacteroides ovatus*, were enriched becoming the prominent species (Figs. 2c and 3c). *Parabacteroides* and *Bacteroides* are some of the genera that had a statistically significant increase in abundance (Table 1C).

### Changes in resistome of mouse gut after antibiotic treatment

Despite the reduction in taxonomy diversity after antibiotic treatment, Fig. 7 and Table 2 show an increased relative abundance for many ARGs. This is against a background of a large number of ARGs declining in relative abundance in samples treated with either amp, cipro or fosfo antibiotics (Supplementary Table 3A-I). For example, *cepA beta-lactamase* sharply increased in relative abundance after the first treatment at 24 h in both cipro and fosfo samples. *cmeB* and *tet37* that were undetectable in control samples show a slight increase in relative abundance after fosfo treatment. Rifampicin resistant ARGs *Nocardia rifampin resistant beta-subunit of RNA polymerase (rpoB2)* and *Bifidobacterium adolescentis rpoB conferring resistance to rifampicin (rpoB)* were detected in cipro samples and their relative abundance in treated samples at 24, 48 and 72 h increased progressively compared to controls. A similar pattern is seen with respect to *ugd* and *mupB* ARGs that were detected in fosfo samples. Like the taxonomy compositions, the resistome compositions (ARG profiles) are generally similar within samples collected at the same time point after treatment with an antibiotic (Fig. 8). Change of MGEs, which have been implicated in the accumulation and dissemination of ARGs (Figs. 9 and 10), were also checked. Relative abundance of *transposases* increased sharply by more than 40% after second (48 h) and third (72 h) treatments with cipro (Fig. 10b). In amp treated 24-h samples *integrases* showed a slight increase (Fig. 9a) but this increase was not sustained in later treatments. Treatment with fosfo caused a complete decline in the relative abundance of *integrases* (Fig. 9c).

**Table 2:** A-C All statistically significant ARGs that are enriched after oral treatment with Ampicillin (A), Ciprofloxacin (B), or Fosfomycin (C)

ARG	Increase/Decrease		
	24 h	48 h	72 h
**A. Ampicillin**			
efrB	–	+	–
LlmA 23S ribosomal RNA methyltransferase	–	+	–
macB	–	–	+
mupA	–	+	–
mupB	–	+	–
patB	–	+	–
Streptomyces rishiriensis parY mutant conferring resistance to aminocoumarin	–	+	–
tetB(46)	–	+	–
tetB(60)	+	+	–
**B. Ciprofloxacin**			
ANT(6)-Ib	+	–	–
arlR	+	–	+
Bifidobacterium adolescentis rpoB conferring resistance to rifampicin	+	+	–
cepA beta-lactamase	+	–	+
cmeB	+	–	–
efrA	+	–	–
efrB	+	–	+
lsaB	–	+	–
macB	+	–	–
mupA	–	+	+
Nocardia rifampin resistant beta-subunit of RNA polymerase (rpoB2)	+	+	+
Streptomyces rishiriensis parY mutant conferring resistance to aminocoumarin	+	–	–
TaeA	–	+	–
tet(W/N/W)	+	–	–
tetA(60)	+	–	+
tetB(P)	+	–	–
tetM	+	–	–
tetW	+	–	–
ugd	+	–	–
vanRC	+	–	+
vanRG	–	+	–
vanRI	–	+	–
vanSC	+	–	–
vanWG	+	–	–
vanYG1	–	+	–
**C. Fosfomycin**			
ANT(6)-Ib	+	–	+
Bifidobacteria intrinsic ileS conferring resistance to mupirocin	–	+	–
catB10	+	–	–
cepA beta-lactamase	+	+	+
cmeB	+	+	+
macB	+	–	–
msbA	+	+	+
mupB	+	+	+
Nocardia rifampin resistant beta-subunit of RNA polymerase (rpoB2)	+	+	–
TaeA	+	+	–
tet37	+	+	+
ugd	+	+	+

For each antibiotic cohort, all bacterial ARGs with a statistically significant increase in relative abundance at any timepoints are listed in tabular form. Plus sign in the column for a timepoint indicates that the ARG count increased after administering treatment at that timepoint and a minus sign indicates a decrease in the count

## Discussion

Although it is known that oral use of antibiotics can disrupt the gut microbiome [22] and could potentially generate antibiotic resistance [8], many factors critical to the gut community-host relationships remain obscure. The scope and complexity of the gut community composition and its impact on host physiology remains an evolving story. The adverse or beneficial results of changes in the taxonomic composition are not well understood. And, the role of the gut microbiome in emergent antibiotic resistance has not been thoroughly characterized. This investigation focused on making preliminary contributions toward defining some of these relationships. Use of a shotgun metagenome sequencing approach over 16S/18S rRNA sequencing allowed for identification of gut microbial species as well as the components of resistome and changes within the resistome in as little as 24 h after antibiotic exposure.

After antibiotic treatment, large populations of bacterial were no longer detectable and the total amount of bacterial genomic DNA was greatly reduced. In some mice, insufficient DNA was acquired for sequencing, thus some numbers in analyses are less than the total number of an experimental group. According to the data generated, the relative abundance of *Acinetobacter* in all control groups was very low, in most cases being undetectable. However, in the groups treated for 48 to 72 h with amp, several species of *Acinetobacter* increased in relative abundance becoming the predominant species (Table 1A, Fig. 3a). Multi species of *Acinetobacter* are pathogens and are very resistant to antibiotics [39]. Emergence of these species with amp treatment could present a resistant infection risk to the individual and to the community. Increase in *Acinetobacter* relative abundance was not seen following cipro and fosfo treatment. This result is consistent with clinical experience with amp. Not only is resistance to amp much more prevalent than resistance to cipro or fosfo across most bacteria, but the sensitivity of *Acinetobacter* to amp is much less than to cipro or fosfo [37, 40, 41]. In groups treated with cipro (24, 48 and 72 h), *Lactobacillus* species exhibited high relative abundance (Table 1B, Fig. 3b) which could be due to the intrinsic resistance harbored by *Lactobacilli* to cipro [42]. Since *Lactobacillus* species are widely used in probiotics [43] and food production [44], their resistance to cipro could be problematic as they could serve as a potential reservoir for antibiotic resistance. Enrichment of *Parabacteroides goldsteinii* and *Bacteroides ovatus* is observed in samples treated with fosfo (Table 1C, Fig. 3c). *B. ovatus* has been implicated in the pathogenesis of irritable bowel diseases (IBD) [45]. Gut community dysbiosis induced by fosfo treatment and subsequent selection of *B. ovatus* could potentially increase the risk of gastrointestinal side effects.

Although the majority of the ARGs that were detected in control groups were undetectable after treatment with antibiotics, a few ARGs were selected for and increased in relative abundance, especially in samples treated with cipro and fosfo (Fig. 7 and Table 2). Ciprofloxacin is a fluoroquinolone, works by binding to DNA gyrase and prevents unwinding of DNA for transcription [46]. ARGs that are selected for after cipro treatment are resistant to a different class of antibiotics. ARGs *rpoB* and *rpoB2* are resistant to rifampicin and *cepA beta-lactamase* confers resistance to beta-lactam antibiotics like ampicillin. Similar results were reported in an earlier genome-wide study (Lázár, 2014) on *Escherichia coli* that mapped out a cross-resistance network for several different classes of antibiotics in which *E. coli* treated with cipro was found to have decreased sensitivity to amp and vice versa [47].

Fosfomycin’s mechanism of action involves suppressing bacterial cell wall synthesis [48] and the ARGs it selected for are resistant to various antibiotics like beta-lactams (*cepA*), tetracycline (*tet37*), mupirocin (*mupB*) and peptide antibiotics (*ugd*). Fosfo also selected for *cmeB* an efflux pump membrane transporter conferring resistance to several different classes of antibiotics. Efflux pumps are a part of intrinsic resistance mechanism of bacteria and are used to decrease levels of different molecules including antibiotics within the cell by pumping them out of bacteria [49, 50]. Evolution of more powerful efflux pumps has been a cause for concern due to their capability to confer cross-resistance to multiple antibiotics. A recent study (Yao, 2016) reported on a new pump *RE-CmeABC* in *Campylobacter jejuni* with increased virulence that can be transferred horizontally [51]. Within the *CmeABC* efflux system, cmeB protein plays a role in identifying and binding to the substrate. Therefore, mutations in cmeB are hypothesized to be responsible for enhanced activity of the *RE-CmeABC* pump [51].

*Transposases* are proteins encoded by transposons that allows the transposons to move from plasmid to chromosome or vice versa and could carry antibiotic resistant gene payload [52, 53]. Interestingly, after treatment with cipro a significant jump in the relative abundance of *transposases* was observed and could signify an increased potential for horizontal gene transfer in the gut community of cipro treated mice. Further studies will need to be designed to verify this result and explore the increase in transposon driven HGT events in cipro treated mice.

This study observed an increase in relative abundance of certain ARGs that were present in control mice as well as new ARGs that were only detectable in treated samples. However, the ability to detect newly emergent resistance genes and mobile genetic elements was limited by the number of known resistance genes within the CARD database [54] and MGEs catalogued in MobileGeneticElementDatabase [55], respectively. This project was not positioned to detect totally novel and previously undescribed ARGs and MGEs. Given that multiple avenues of genetic material exchange have been recognized in bacteria, the increased risk for this concentration of genetic resistance to move to other bacteria in either the gut community or the host’s environment merits future investigation. Moreover, these findings were the result of a 3-day treatment regimen in experimental animals. Other regimens and durations of exposure may have a different impact on the taxonomic composition and resistome and will require further study to characterize the off-target effects of antibacterial treatment.

## Conclusions

In summary, oral antibiotic therapy caused a longitudinal decrease in the overall gut microbial diversity along with enrichment of specific taxa and ARGs in UTI mouse model. Results from this model also point to a selection pattern with respect to ARGs that result in emergence of cross-resistance to multiple classes of antibiotics after 24 to 72 h of treatment with cipro and fosfo.

## Methods

### Animal model and antibiotic exposure

All animal studies were conducted in 8–10 weeks old female Balb/c mice purchased from Taconic Farms (Derwood, MD), housed and cared for via the Guide for the Care and Use of Laboratory Animals 8th Edition, under an Institutional Animal Care and Use Committee approved protocol in the AAALAC accredited Animal Program of the White Oak Federal Research Center. To limit the individual variation of the gut microbiome in experimental groups, the same strain, sex and age mice were obtained from the same vendor and the same location in the vendor facility. Mice were maintained on the same diet with social housing conditions for each experiment. Contamination from the sampling process and equipment were considered and procedures established to mitigate bacterial contamination. Within these experiments, control mice demonstrated minimal variation in microbiome composition despite using different batches of mice for each antibiotic exposure (Fig. 1 and Supplementary Fig. 1). Three cohorts of 20 mice each were selected, and each cohort contained 12 treatment and 8 control group animals. The control group was split into two sub-groups – naïve and infection with four animals in each group. Naïve group animals were neither infected nor treated with antibiotic and infection group animals were infected but not treated with antibiotic. Mice were randomly allocated to treatment groups that were subsequently housed together in groups of either 4 (controls) or 6 (treated) mice. The ascending, unobstructed UTI model was used as previously described [56, 57] with slight modification using CFT073 uropathogenic *E. coli* acquired from ATCC (Manassas, VA, U.S.). Antibiotics, ampicillin trihydrate (Sigma, St. Louis, MO, U.S.) 200 mg/kg in 0.1 M HCl, ciprofloxacin 5% oral suspension (Bayer HealthCare, Whippany, NJ, U.S.) 50 mg/kg and Monurol® (Fosfomycin tromethamine, Forest Pharmaceutical, INC, St Louis, MO, U.S.) 1000 mg/kg in water were all administered orally. Food and water were allowed ad libitum. Literature was used to design in-house pharmacokinetic studies [58–60] and results from those studies were used to select dosing concentrations and intervals for the present study in which amp and cipro were given twice daily at 8-h intervals and fosfo was given once daily for three consecutive days. Fecal samples were collected at euthanasia from the distal ileum and proximal colon after 24 h (1 day), 48 h (2 days) and 72 h (3 days) of treatment. After 72 h, all control group animals were euthanized, and fecal samples harvested. Humane euthanasia was achieved by exsanguination under isoflurane anesthesia and pneumothorax. The details of the experiment design are provided in Table 3.

**Table 3:**
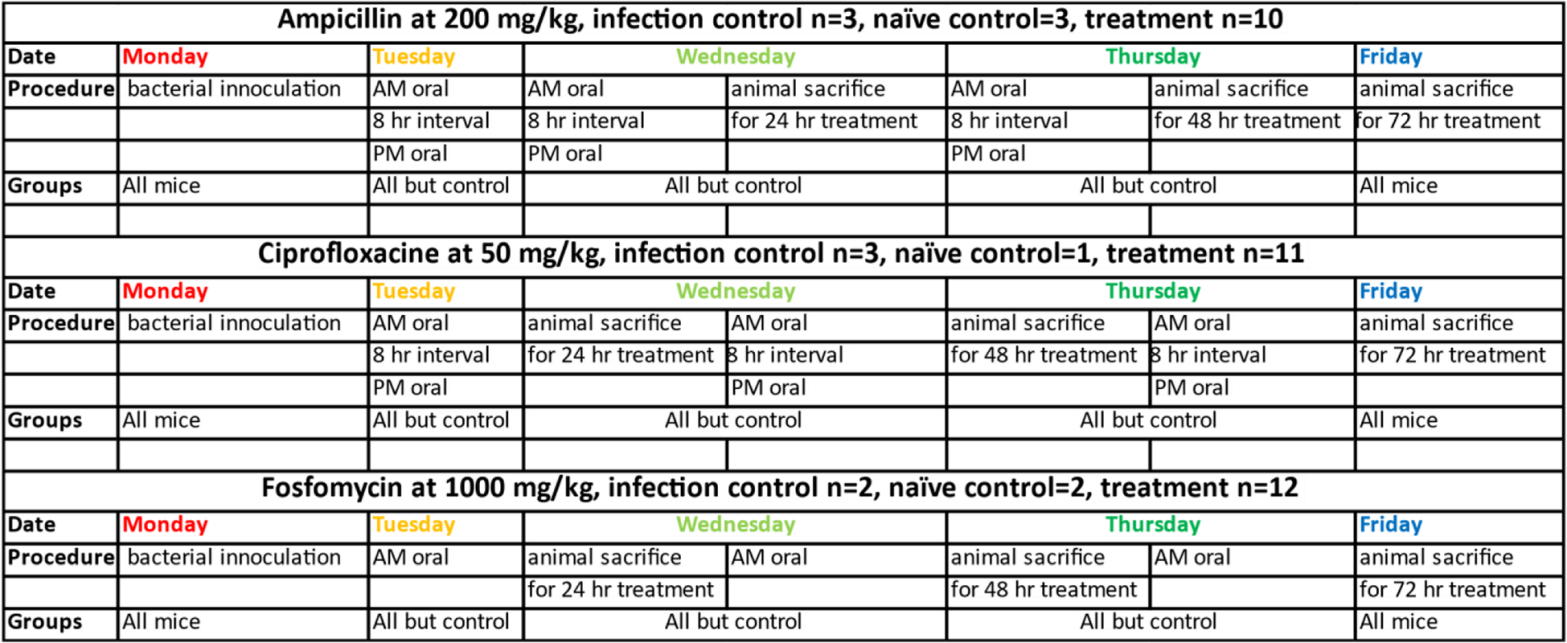
Experimental design

Details about number of animals present in each cohort and time points at which the antibiotic was administered, and samples harvested

### Genomic DNA extraction and metagenome shotgun sequencing

After 1, 2, or 3 days of treatment with fosfo, cipro, or amp, stool samples were obtained from the intestinal tract at sacrifice. Genomic DNA was extracted using QIAamp DNA Stool Mini kit (Qiagen, MD), modified in the first step using Tungsten Carbide Beads 3 mm (Qiagen, Str. 1, 40,724 Hilden, Germany) in sample disruption with TissueLyser LT (Qiagen, MD) for high-speed shaking at 50hz for 1 min. Stool samples were then homogenized in lysis buffer and heated for 10 min at 70 °C and PCR Inhibitors were removed by inhibitEX Tablets (Qiagen, MD). Supernatants were enzymatically digested using proteinase K (Qiagen, MD) for 5 min at 70 °C. Genomic DNA was purified using QIAamp mini spin column. DNA quality was evaluated with an Agilent 2100 Bioanalyzer A260/280 and quantified with a Qubit 4 Fluorometer (Thermo Fisher Scientific, NY). Seventy-five nanograms of genomic DNA was used for library preparation following the Nextera DNA library prep reference guide 2016. Tagmented DNA was purified using DNA Clean & ConcentractorTM-5 (Zymo research, Irvine, CA) and library DNA was purified by AMPure XP beads (Beckman Coulter Life Science, Brea, CA). Incubation and thermal cycling were performed on a Thermo Scientific™ Arktik™ Thermal Cycler (Thermo Fishere Scientific, Waltham, MA). Size range was assessed on Agilent 2100 bioanalyzer (Agilent Technology, Santa Clara, CA) with High Sensitivity DNA Analysis Kit (Agilent Technology, Santa Clara, CA) and quantified on the Qubit 4 Fluorometer. Library pools for sequencing according to NextSeq System Denature and Dilute Libraries Guide (Illumina, 2016). Metagenome sequencing was performed using NextSeq 500 sequencing system ((Illumina, San Diego, CA) with paired-end (Ampicillin & Ciprofloxacin) or unpaired-end (Fosfomycin) shotgun sequencing [61]. Not all animals yielded usable sequencing data due to premature loss of the animal, limited stool, or insufficient amount of extracted DNA. The number of useable samples in the sequencing analyses were as follows: Ciprofloxacin: there were a total of four control samples (3 naïve plus 1 infection), the groups harvested after 24 and 48 h of treatment were comprised of 4 samples each, while the group sacrificed after 72 h of treatment contained only 3 samples; Fosfomycin: the control group contained four animals (2 naïve plus 2 infection), the treatment groups that are harvested at 24, 48, and 72 h of treatment each contained 4 samples; Ampicillin: there were a total of 6 (3 naïve plus 3 infection) controls, the groups sacrificed after 24 and 72 h of treatment contained 3 samples each while the group sacrificed after 48 h of treatment contained 4 samples. Samples were never pooled in any of the experiments. Principle Coordinate Analysis (PCoA) plot (Supplementary Fig. 1) based on Bray–Curtis dissimilarity of ARG abundances of all samples from three cohorts demonstrates that both naïve and infection control group samples are similar to each other as they congregate together in a single group. Due to this reason, during downstream analysis, naïve and infection control groups within each cohort were considered as single group of control samples.

### Metagenomic analysis

Illumina bcl2fastq [62] version 2.18.0.12 was used to demultiplex and trim adapter sequences. Read quality analysis was done with FastQC version 0.11.3 [63] and quality trimming was performed using Trimmomatic version 0.39 [64] with parameters SLIDINGWINDOW:4:15 MINLEN:30. BWA version 0.7.16 [65] was used to remove the mouse DNA by mapping reads against mouse genome (GRCm38). Unmapped SAM files containing bacterial reads were converted to FASTQ format using SamToFastq command in Picard Tools version 2.1.1 [66]. Samples with close to 10 million bacterial reads were retained for downstream analysis. Sample information and read counts at different stages of the process are shown in Supplementary Table 1. Metagenome sequencing data have been deposited in the National Center for Biotechnology Information (NCBI) BioSample Submission Portal as Bioproject PRJNA478457 and SRA accession number SRP152866.

makeblastdb command from NCBI BLAST+ version 2.3 [67] was used to create a custom BLAST database from Comprehensive Antibiotic Resistance Database [54] protein homolog model version 3.0.3 (CARD) FASTA file and blastx command was used to characterized the resistome by mapping reads against this custom BLAST database. BLAST results with an E-value cut-off of 10^− 5^, identity ≥80% and query coverage ≥50% were retained and counted to generate a list of ARG abundances. MGE counts were determined by mapping bacterial reads against MobileGeneticElementDatabase [55] using Bowtie2 version 2.3.2 [68] with parameters -D 20 -R 3 -N 1 -L 20 -i S,1,0.50. If both forward and reverse reads of a paired-end sequence map to the same MGE, then it was counted as one; if they both map to different MGEs, they were counted towards those specific MGEs [69]. MGE counts in each sample were aggregated based on MGE type.

### Taxonomic analysis

16S rRNA reads were extracted and taxonomic counts were generated from shot-gun metagenomic samples using Metaxa2 [35] version 2.2 with default settings. The classification database used by Metaxa2 was based on SILVA SSU database [70] version 132 reference database. Metaxa2 classifies sequences based on Hidden Markov models (HMMs) and requires generation of HMM profiles and custom BLAST database. For this purpose, Metaxa2 companion tool called Metaxa2 Database Builder [71] was used to generate a custom Metaxa2 classification database in conserved mode with the following parameters --cutoffs 0,75,78.5,82,86.5,94.5,98.65 --full_length 0 --plus T. Metaxa2 output was further processed using Metaxa2 Diversity Tools [72] (metaxa2_ttt and metaxa2_dc) to generate genus abundance matrix for each cohort.

Metaphlan2 profiles the compositional species of gut microbial communities with accurate organismal relative abundance relying on ~ 1 M unique clade-specific marker genes from metagenomic shotgun sequencing data. Viruses and other organism were removed and just bacteria species were kept, and abundance of bacteria was adjusted for further analysis. A heatmap was created for each antibiotic using R with ggplot2 package to demonstrate clustering of samples based on the similarity of compositional species profiles (log-transformed relative abundance across all identified species). Hierarchical clustering was performed and a dendrogram was created using unweighted pair group mean with arithmetic mean (UPGMA) algorithm (53, 54, 55). The order of the listed bacterial species in each heatmap of the Fig. 2 was generated by clustering the species across samples and was unique for each antibiotic. These rosters were created to best depict the clustering (or similarity) of samples. In subsequent comparisons (the remaining heatmaps), a roster was created that captured all species identified across all antibiotics. The order of this roster was standardized to allow comparisons across antibiotics as well as between treatments and controls.

### Statistical analysis

Analyses were performed in R v.3.6.1 using metagenomeSeq [73], edgeR [74], ggplot2 [75] and vegan [76] packages. Data cleanup was performed by removing low-abundance features (ARG, MGE and 16S counts) that were not present in at least two samples and/or contained a count of less than 10 per sample. Samples were retained for analysis if they contain positive counts for two or more features. Data normalization to account for sequencing depth was performed by dividing the abundance counts in each sample by counts-per-million factor which was obtained by dividing the total number of sequences in that sample by 1,000,000.

**Fig. 4:**
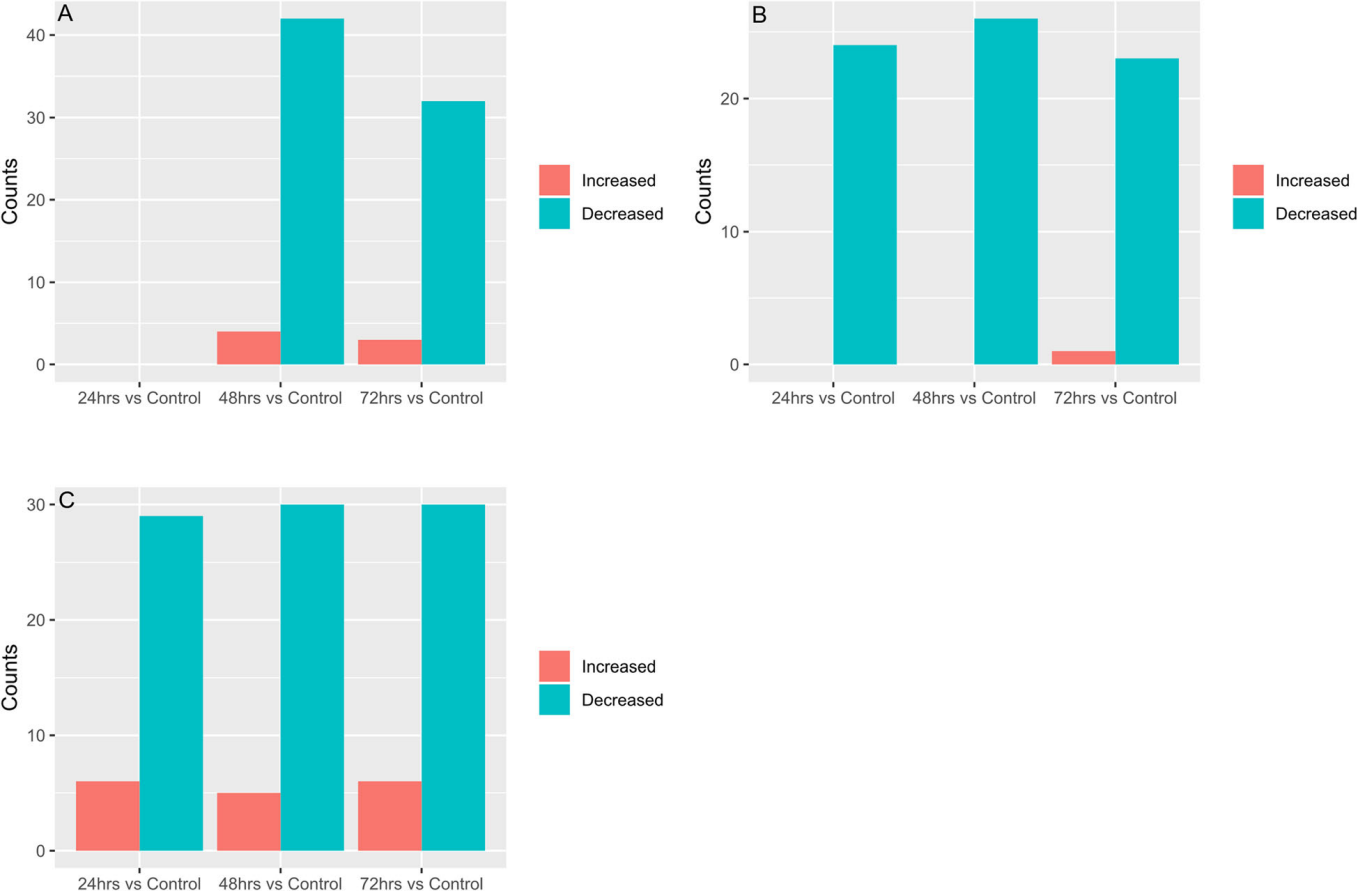
**a**-**c** Change in the number of statistically significant genera detected in treatment samples compared to control samples based on 16S rRNA analysis. The 16S rRNA sequences were re-constructed from metagenome shotgun and taxonomy was assigned using Metaxa2 and SILVA database. metagenomeSeq used to find bacterial genera that were statistically significant, differentially represented between control and treatment time points (24, 48, and 72 h) after oral treatment with Ampicilin (**a**), Ciprofloxacin (**b**), or Fosfomycin (**c**). Green bars represent number of genera at each time point (24, 48, and 72 h) that exhibited a decreased relative abundance compared to control. Red bars represent number of genera that exhibited increased relative abundance. A list of these genera for each cohort/treatment combination along with their fold change values is provided in Supplementary Information Table 2a-i

**Fig. 5:**
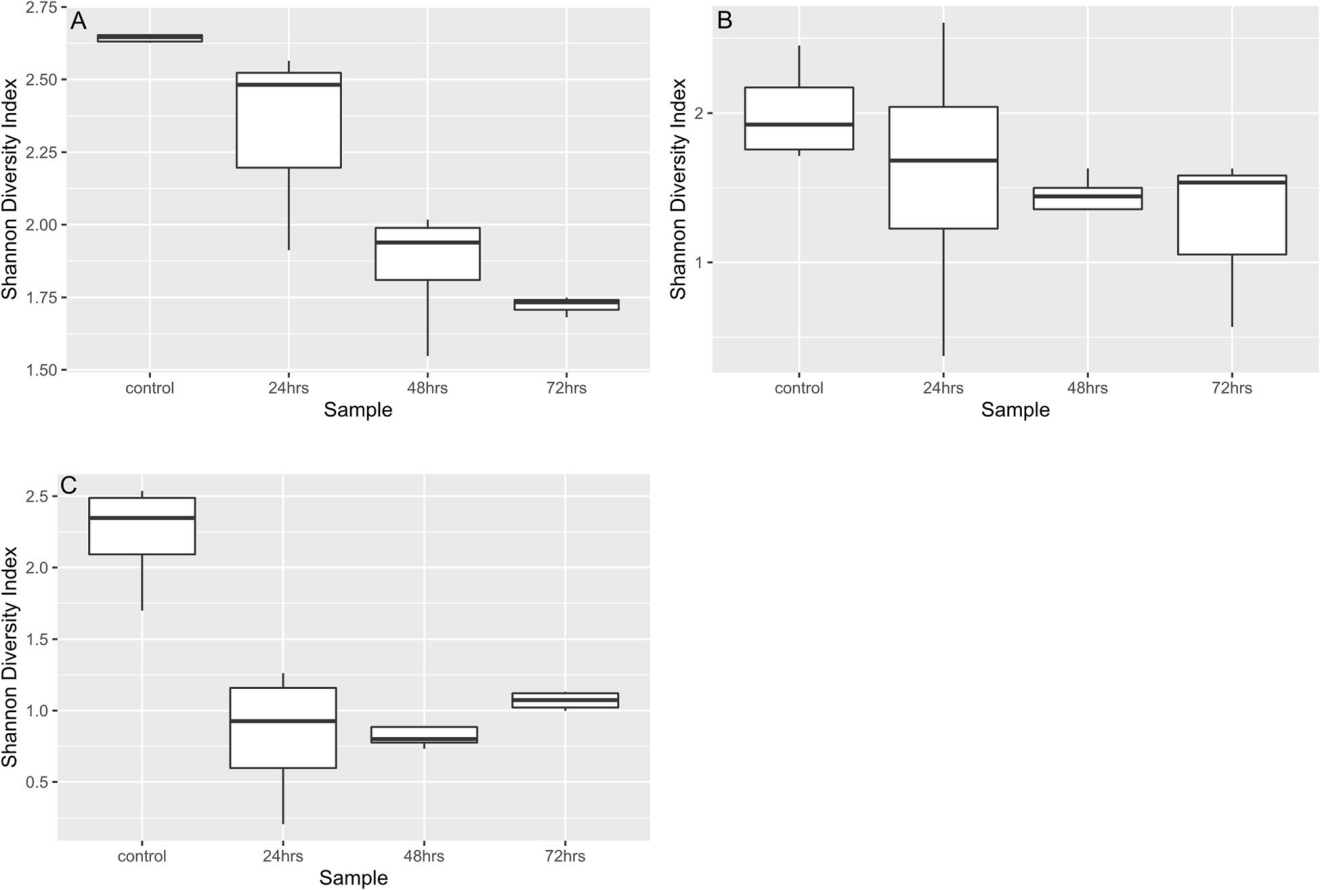
**a**-**c** Longitudinal change of microbiome diversity after antibiotic treatment. Time-dependent change of the microbiome diversity, calculated as the Shannon diversity index based on 16S rRNA reads found by Metaxa2, is shown for the Ampicillin (**a**), Ciprofloxacin (**b**), or Fosfomycin (**c**) cohorts. The diversity index of controls for Ampicillin and Fosfomycin are higher compared to ciprofloxacin. Ampicillin treatment caused a gradual decrease in diversity whereas Fosfomycin treatment caused a steep decline in diversity at 24 h and a small but gradual recovery. Ciprofloxacin treated samples at 24 h were more diverse than control, but the diversity was reduced at 48 and 72 h

**Fig. 6:**
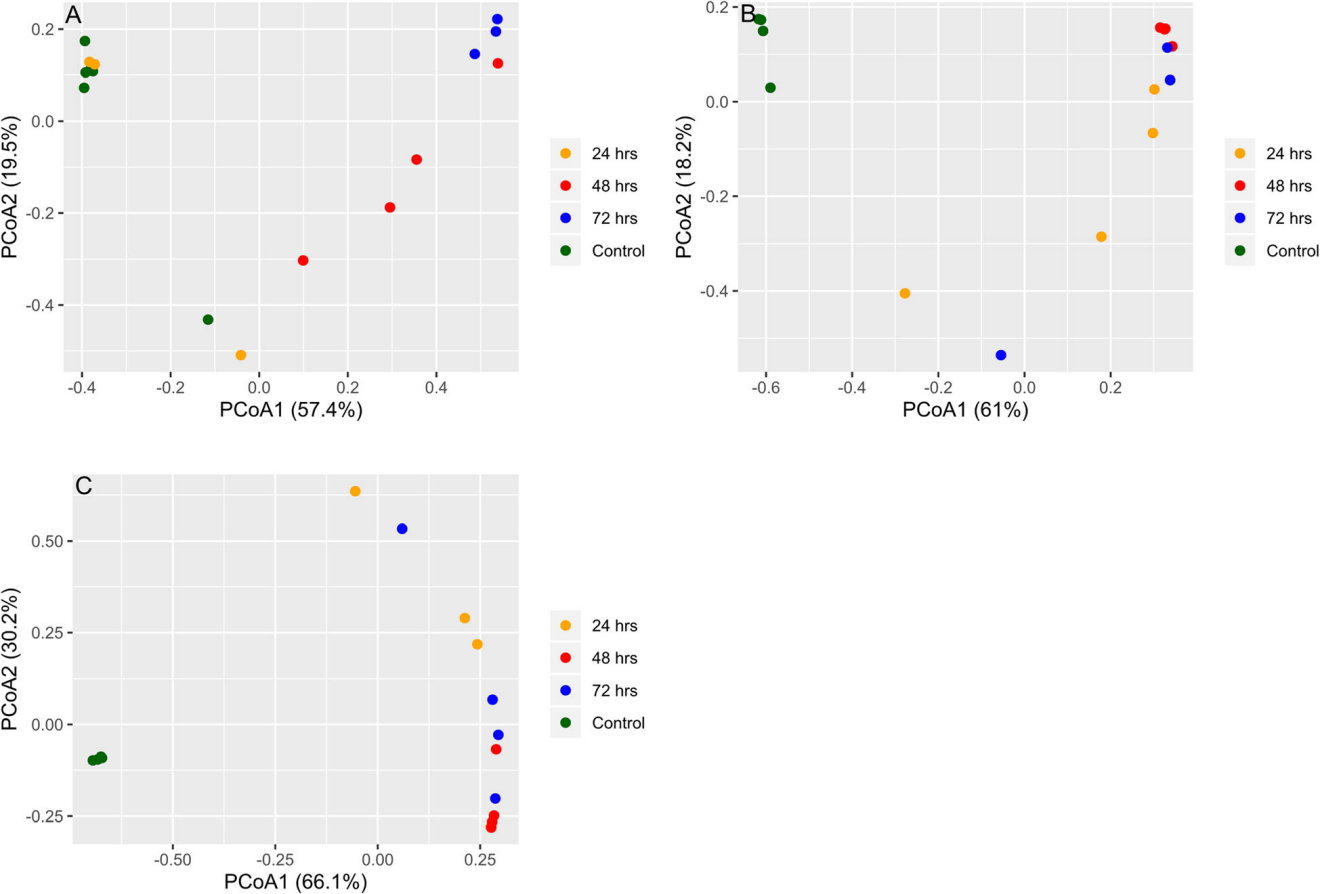
**a**-**c** Principle Coordinate Analysis (PCoA) reveals the time-dependent shift of metagenome profiles after oral treatment with Ampicillin (**a**), Ciprofloxacin (**b**), or Fosfomycin (**c**). For each antibiotic cohort, the bacteria genera identified from each sample (solid dots) were subject to PCoA and the first and second principle coordinates are shown as X and Y axis, respectively. Within all three cohorts, there was a general trend in the way samples grouped together. Control samples grouped together and away from treated samples (24, 48, 72 h) indicating a change in genus profiles after treatment. The only exception being ampicillin where two of the 24 h samples grouped with controls

Quasi-likelihood F-test (glmQLFTest function) was performed on the normalized 16S rRNA abundance data using edgeR [74]. Within each cohort, differentially abundant genera for control vs treatment (24, 48 and 72 h) groups are considered statistically significant if Benjamini–Hochberg false discovery rate is below 0.05*.* Bar plots were plotted for each cohort to depict the number of genera that have a statistically significant fold change between control and treatment samples in either direction (Fig. 4). A list of these statistically significant genera is available in Supplementary Information Table 2a-i. Shannon diversity index was calculated using vegan [76] package box plots (Fig. 5) depicting the change in Shannon diversity were plotted for each of the three cohorts. The shift in diversity profiles between control and treatment groups were examined using principal coordinates analysis (PCoA) based on Bray-Curtis dissimilarity values (Fig. 6).

**Fig. 7:**
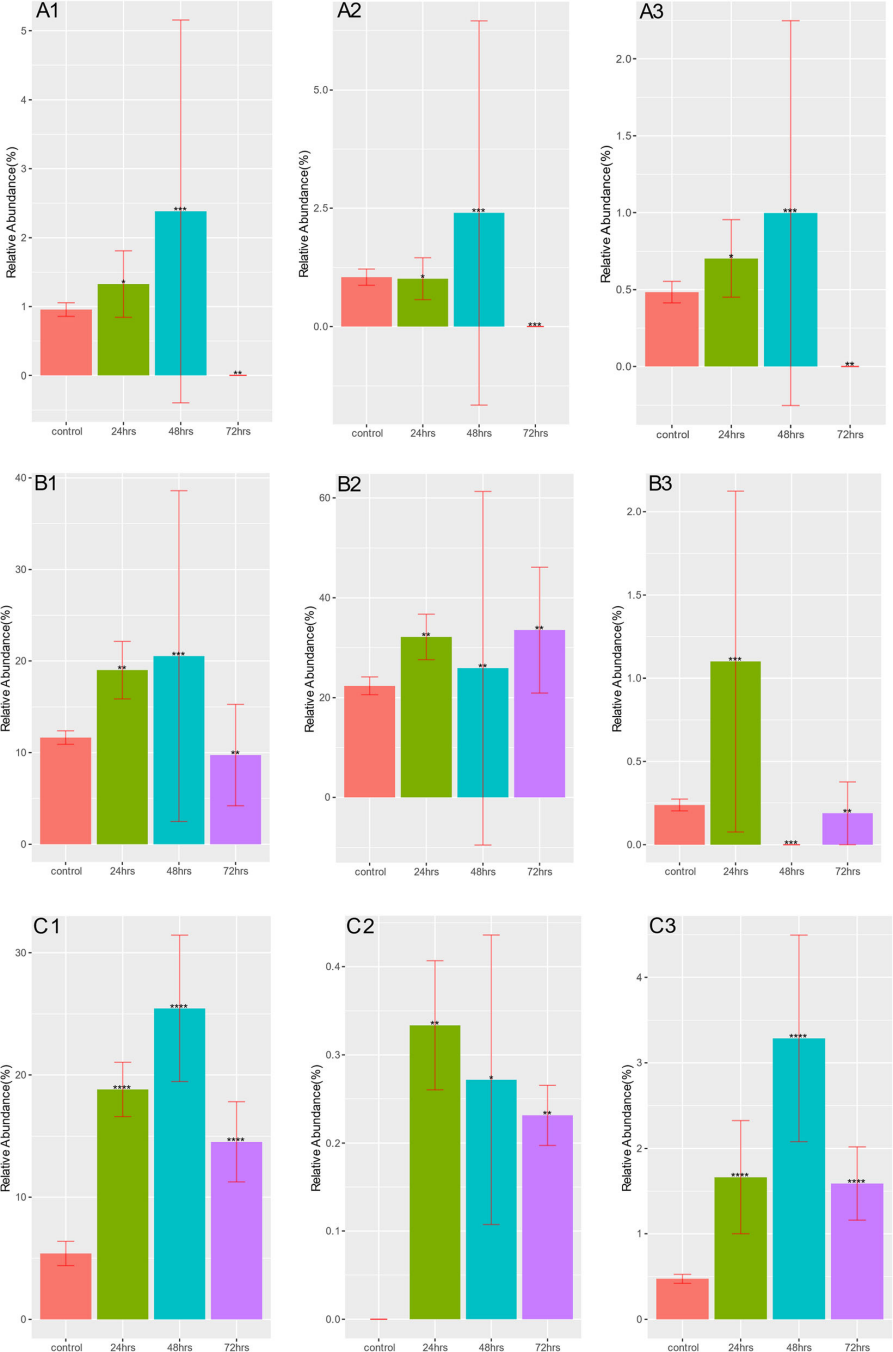
**A**-**C** Relative abundance of enriched ARGs (CARD database; 36) that are statistically significant and differentially represented at all three timepoints (24, 48, or 72 h) in samples treated with antibiotics. Relative abundance of ARGs that were enriched and had a statistically significant change at any of the three timepoints (24, 48, or 72 h) after oral treatment with Ampicillin (A1, LImA23s ribosomal RNA methyltransferase; A2, efrB, a part of the EfrAB efflux pump; A3, parY mutant conferring resistance to aminocoumarin), Ciprofloxacin (B1,Bifidobacterium adolescentis rpoB conferring resistance to rifampicin; B2, Nocardia rifampin resistant beta-subunit of RNA polymerase (rpoB2); B3, Streptomyces rishiriensis parY mutant conferring resistance to aminocoumarin), or Fosfomycin (C1, ugd, required for the synthesis and transfer of 4-amino-4-deoxy-L-arabinose (Ara4N) to Lipid A; C2, cmeB, the inner membrane transporter of the CmeABC multidrug efflux complex; C3, mupB, an alternative isoleucyl-tRNA synthetase conferring resistance to mupirocin) are shown. The height of each bar corresponds to the average relative abundance of ARG for that specific timepoint. The relative abundance was calculated as the percentage of reads mapped to each ARG within the sample. Statistically significant change of relative abundance was defined as those pairwise comparisons between control and any time points with an FDR < =0.05 (* FDR < =0.05, ** FDR < = 0.01, *** FDR < = 0.001, **** FDR < = 0.0001). Only representative ARGs are shown. The full list can be found in Table 2

**Fig. 8:**
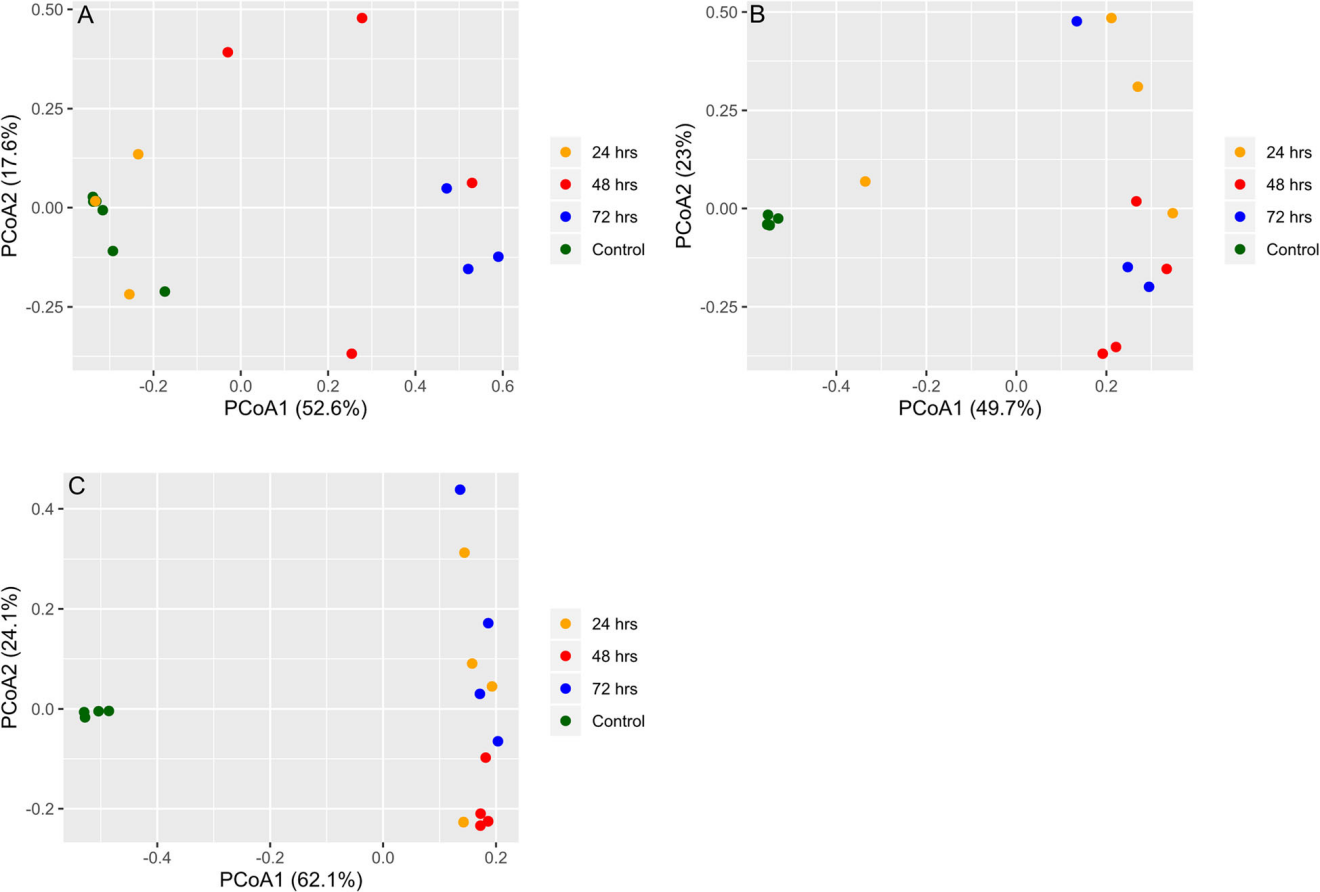
**a**-**c** Principle Coordinate Analysis (PCoA) based on Bray–Curtis dissimilarity of ARG abundances between samples reveals the time-dependent shift of antibiotic resistant gene (ARG) profiles after oral treatment with Ampicillin (**a**), Ciprofloxacin (**b**), or Fosfomycin (**c**). For each antibiotic cohort, the first and second principle components are shown on X and Y axis, respectively. For all three cohorts, there was a general trend that control samples grouped together along either the X and/or Y axis, indicating control samples had similar profiles of ARG. All treatment samples for Ciprofloxacin and Fosfomycin clustered along the Y axis away from control samples indicating a dramatic shift in the ARG profiles compared to control samples. On the other hand, Ampicillin treated samples showed a gradual shift in ARG profiles with 24-h treatment samples clustering along with controls, 48-h samples scattered in the middle and 72-h samples clustered at the other end of the plot

**Fig. 9:**
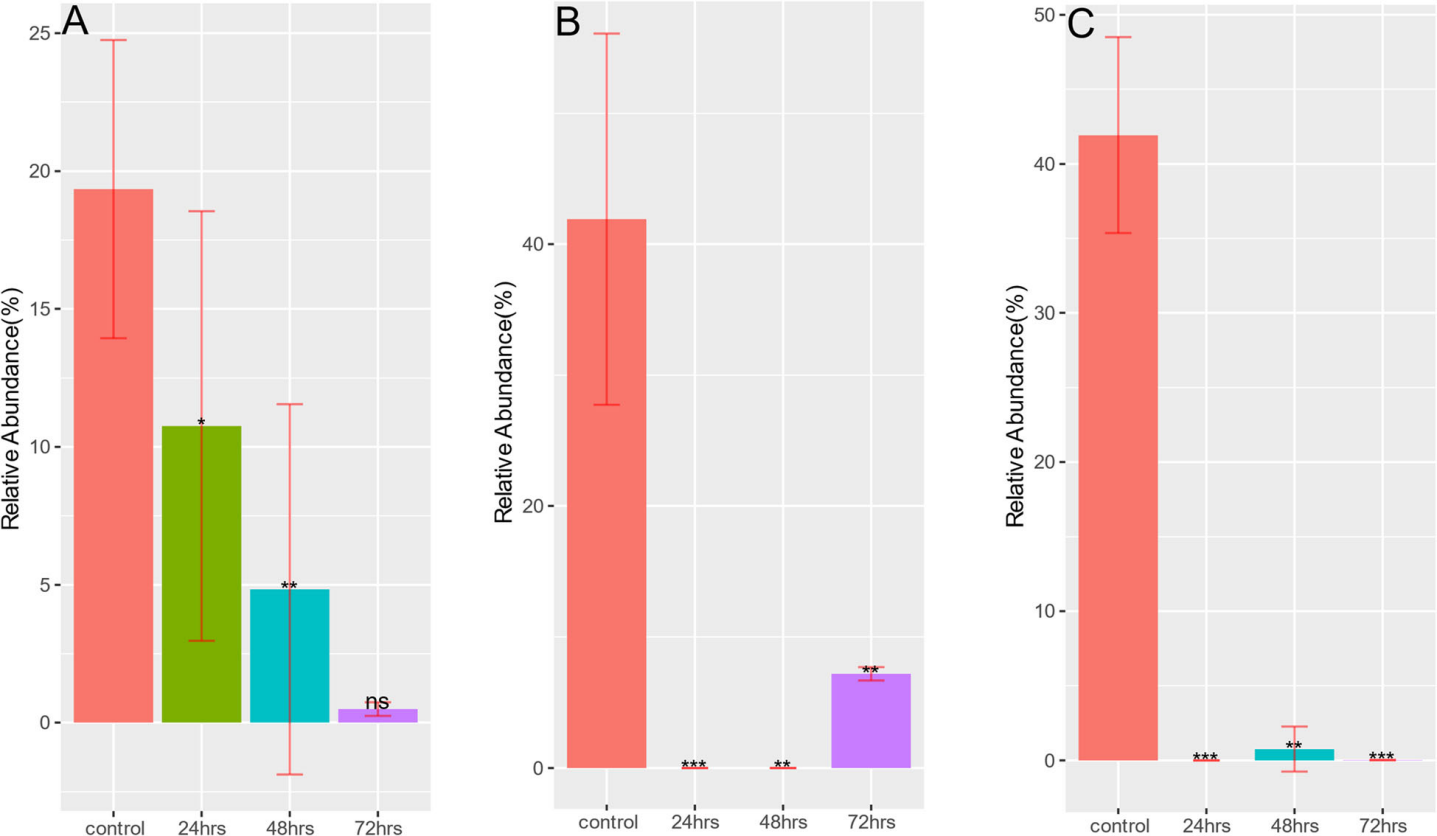
**a**-**c** Statistically significant change in relative abundance of MGE integrase between control and treatment groups. Relative abundance of MGE integrase that had statistically significant change after Ampicilin (**a**), Ciprofloxacin (**b**), or Fosfomycin (**c**) treatments. The height of each bar corresponds to the average relative abundance of integrase for that specific timepoint. The relative abundance was calculated as the percentage of reads mapped to each MGE within the sample. Statistically significant change of relative abundance was defined as those pairwise comparisons between control and 24, 48, and 72 h time points with an FDR < =0.05 (* FDR < =0.05, ** FDR < = 0.01, *** FDR < = 0.001, **** FDR < = 0.0001, ns FDR > 0.05)

**Fig. 10:**
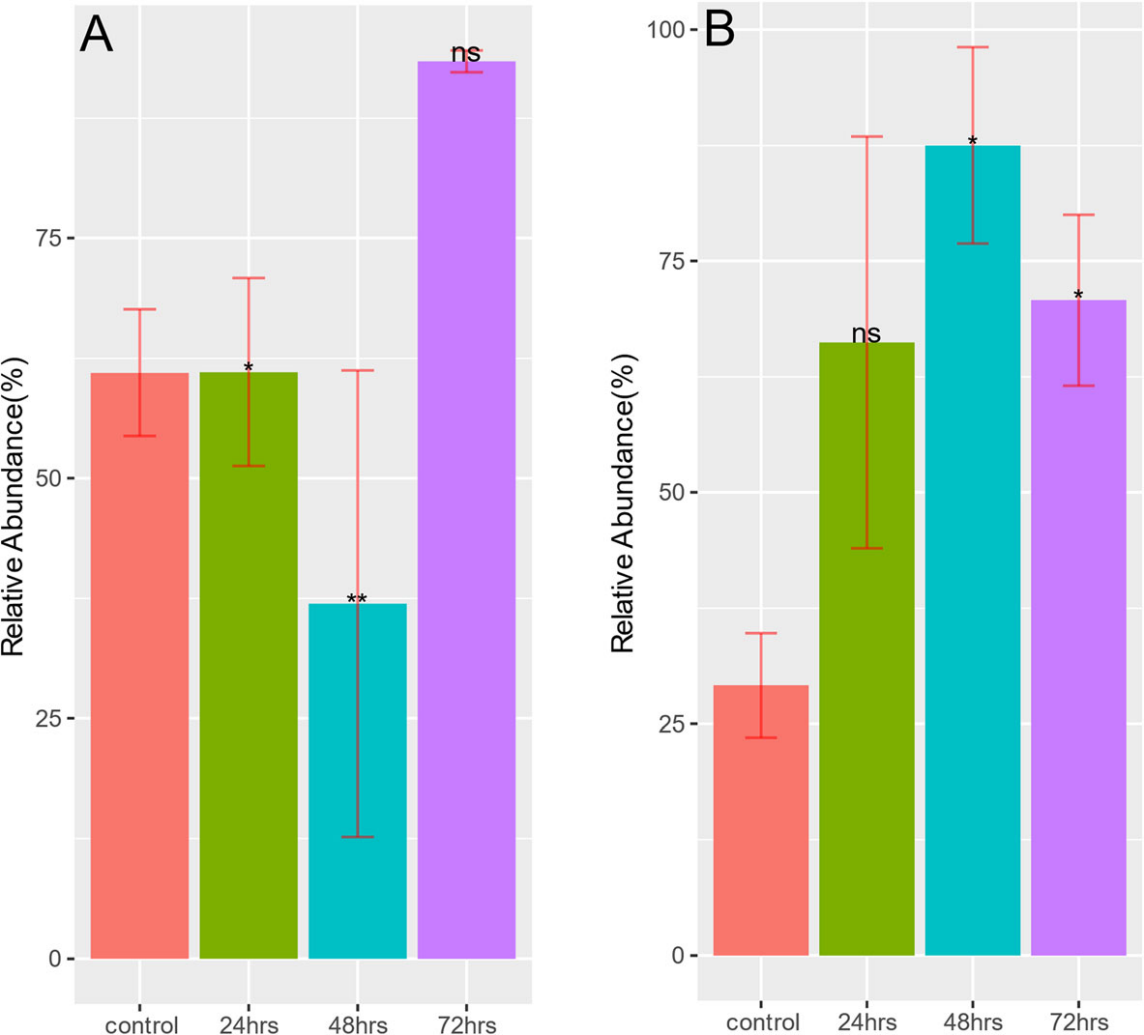
**a** and **b** Statistically significant change in relative abundance of MGE transposase between control and treatment groups. Relative abundance of MGE transposase that had statistically significant change after Ampicilin (**a**) and Ciprofloxacin (**b**) treatments. No significant change was detected with Fosfomycin treatment. The height of each bar corresponds to the average relative abundance of transposase for that specific timepoint. The relative abundance was calculated as the percentage of reads mapped to each MGE within the sample. Statistically significant change of relative abundance was defined as those pairwise comparisons between control and 24, 48, and 72 h time points with an FDR < =0.05 (* FDR < =0.05, ** FDR < = 0.01, *** FDR < = 0.001, **** FDR < = 0.0001, ns FDR > 0.05)

To test for differentially abundant ARGs and MGEs, fitZig function in MetagenomeSeq [73] package was used with all four groups (control, 24, 48 and 72 h) as input to the model. fitZig uses a zero-inflated Gaussian mixture model and expectation-maximization algorithm to estimate differential abundance [73]. Contrasts were made between control and treatment groups (24, 48 and 72 h) to obtain a list of statistically significant (FDR < 0.05) features for each of the three comparisons. For each cohort, top 5 ARGs based on fold-change across all three comparison groups were picked and their relative abundance plotted at different time points of treatment (Fig. 7). Using ARG abundance data, Bray-Curtis dissimilarity indices were calculated with vegan [76] package and principal coordinates analysis (PCoA) was performed to show the shift in diversity across control and treatment groups (Fig. 8). Bar plots (Figs. 9 and 10) depicting changes in relative abundance of statistically significant (FDR < 0.05) MGEs between control and treatment groups (24, 48 and 72 h) within each cohort were plotted.

## Supplementary information

**Additional file 1: Supplementary Figure 1.** Principle Coordinate Analysis (PCoaA) based on Bray–Curtis dissimilarity of ARG abundances for all sample groups across all three cohorts Ampicillin (A), Ciprofloxacin (B), and Fosfomycin (C). **Supplementary Table 1.** Sample read counts. **Supplementary Table 7.** Shared species.

**Additional file 2: Supplementary Table 2.** Statistically significant genera between control and treatment groups.

**Additional file 3: Supplementary Table 3.** Statistically significant ARGs between control and treatment groups.

**Additional file 4: Supplementary Table 4.** Ampicillin species relative abundance.

**Additional file 5: Supplementary Table 5.** Ciprofloxacin species relative abundance.

**Additional file 6: Supplementary Table 6.** Fosfomycin relative abundance.

## Data Availability

All of the raw sequencing reads were deposited in Sequence Read Archive (sra@ncbi.nlm.nih.gov) with an accession number SRP152866. Other data and information can be obtained via request to the corresponding author.

## Abbreviations

cipro: Ciprofloxacin
fosfo: Fosfomycin
amp: Ampicillin
MGE: Mobile genetic elements
ARG: Antibiotic resistance gene
HGT: Horizontal gene transfer
