# Supplementary material for: The effect of antibiotics on the gut microbiome: a metagenomics analysis of microbial shift and gut antibiotic resistance in antibiotic treated mice
Source: BMC Genomics. 2020 Mar 30;21:263. doi: 10.1186/s12864-020-6665-2 (PMC7106814; doi:10.1186/s12864-020-6665-2)
Supplement: Supplementary file 1 — Additional file 1: Supplementary Figure 1. Principle Coordinate Analysis (PCoaA) based on Bray–Curtis dissimilarity of ARG abundances for all sample groups across all three cohorts Ampicillin (A), Ciprofloxacin (B), and Fosfomycin (C). Supplementary Table 1. Sample read counts. Supplementary Table 7. Shared species. [file 12864_2020_6665_MOESM1_ESM.docx]

**Supplementary Information:**

**
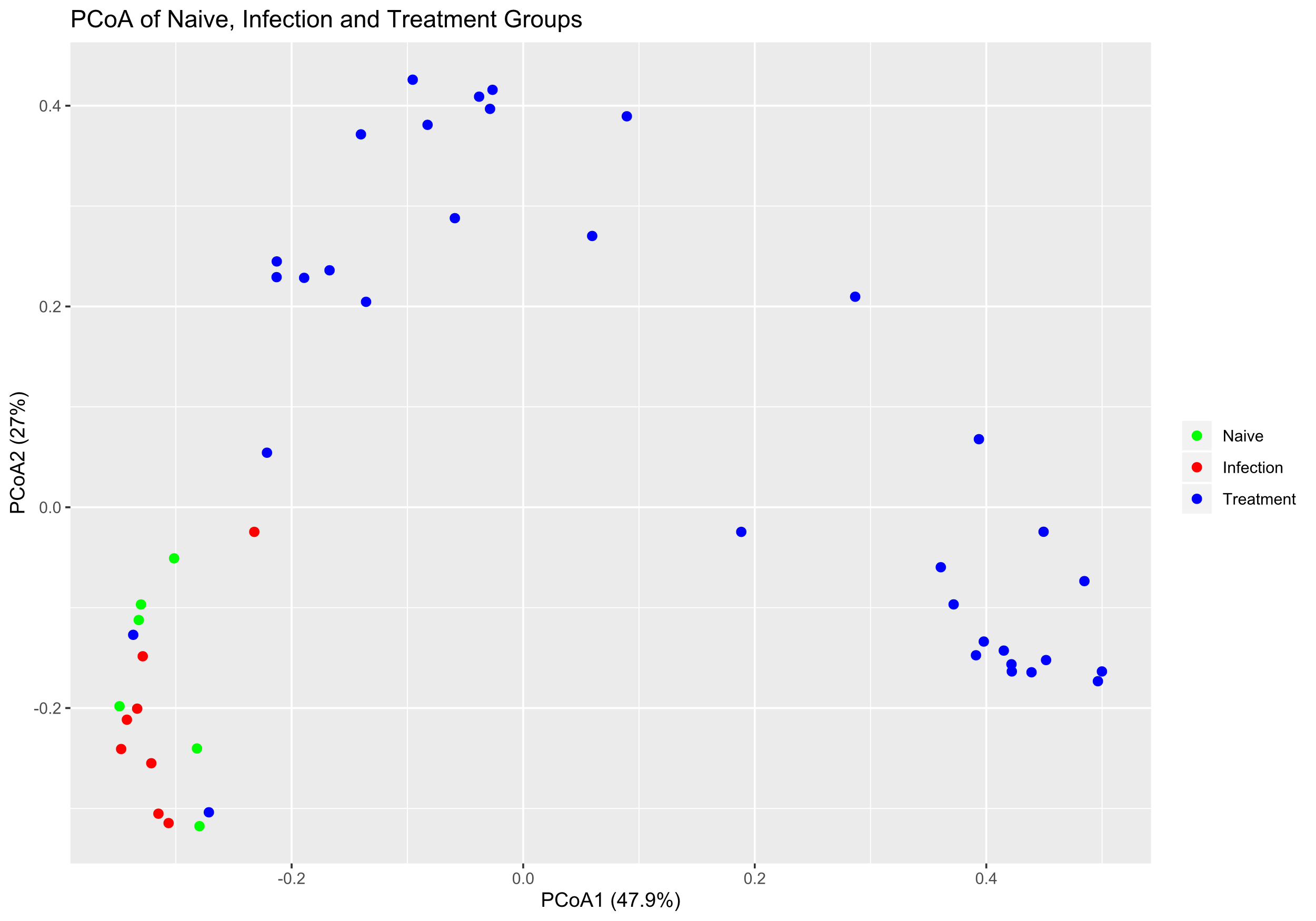
**

*Supplementary Figure 1. Principle Coordinate Analysis (PCoaA) based on Bray–Curtis dissimilarity of ARG abundances for all sample groups across all three cohorts Ampicillin (A), Ciprofloxacin (B), and Fosfomycin (C).*

*First and second principle components are shown on X and Y axis, respectively. All naïve and infection control samples clustered together, indicating that they have similar ARG profiles. Naïve and infection control mice clustered together along y-axis away from most treated mice with an exception of two 24-hour amp treated mice that clustered with controls.*

*
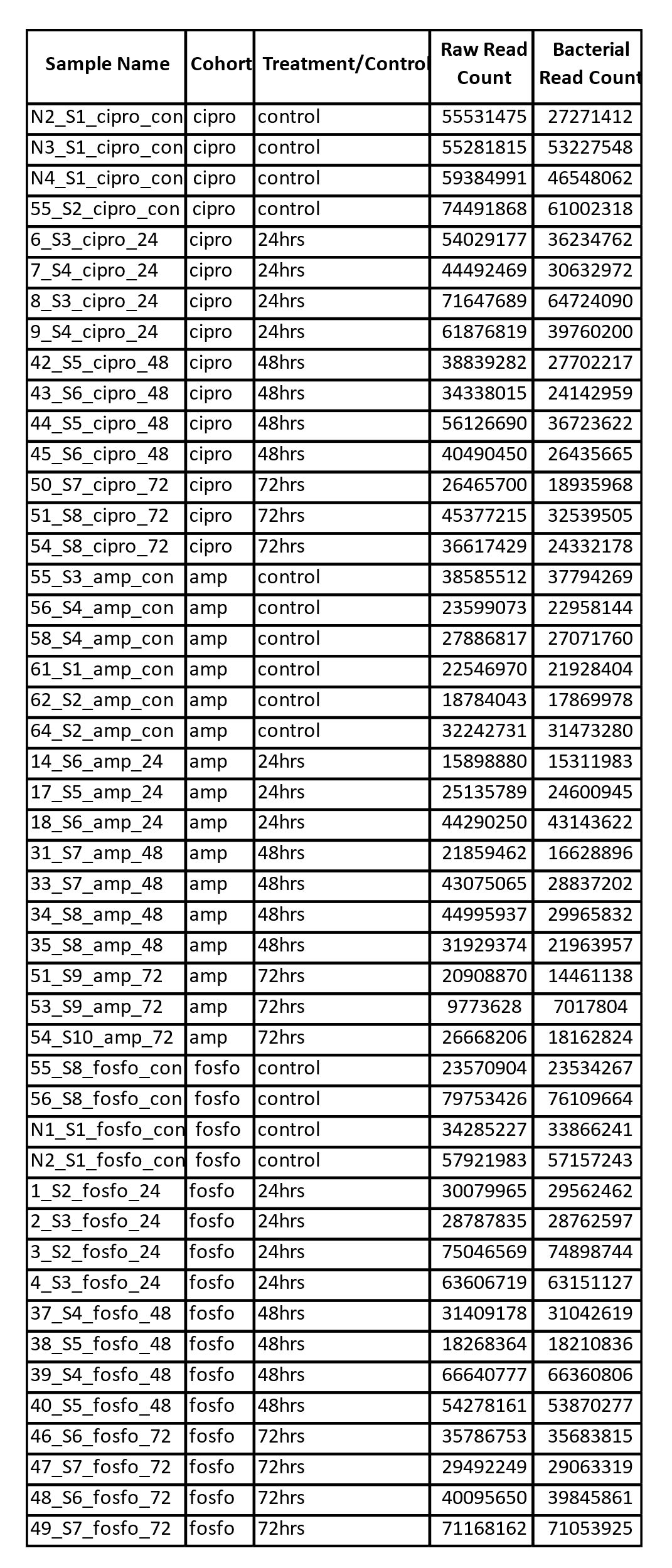
*

*Supplementary Table 1. Sample read counts

List of samples across all cohorts and treatment groups with corresponding raw and bacterial read counts.*

See file: ***Supplementary Info Table 2 A-I - Statistically Significant Genera.xlsx***

*Supplementary Table 2. Statistically significant genera between control and treatment groups.*

*List of statistically significant (FDR < 0.05) genera for each comparison (24 hrs vs control, 48 hrs vs control, 72 hrs vs control) done using EdgeR for all three cohorts (amp, cipro and fosfo).*
See file: ***Supplementary Info Table 3 A-I - Statistically Significant ARGs.xlsx***

*Supplementary Table 3. Statistically significant ARGs between control and treatment groups.

List of statistically significant (FDR < 0.05) ARGs for each comparison (24 hrs vs control, 48 hrs vs control, 72 hrs vs control) done using MetagenomeSeq for all three cohorts (amp, cipro and fosfo).*

See file: ***Supplementary Info Table 4 - Species Relative Abundance - Ampicillin.xlsx***

*Supplementary Table 4. Ampicillin species relative abundance

Relative abundance of species detected by Metaphlan2 in amp samples.*

See file: ***Supplementary Info Table 5 - Species Relative Abundance - Ciprofloxacin.xlsx***

*Supplementary Table 5. Ciprofloxacin species relative abundance

Relative abundance of species detected by Metaphlan2 in cipro samples.*

See file: ***Supplementary Info Table 6 - Species Relative Abundance - Fosfomycin.xlsx***

*Supplementary Table 6. Fosfomycin relative abundance

Relative abundance of species detected by Metaphlan2 in fosfo samples.*

*
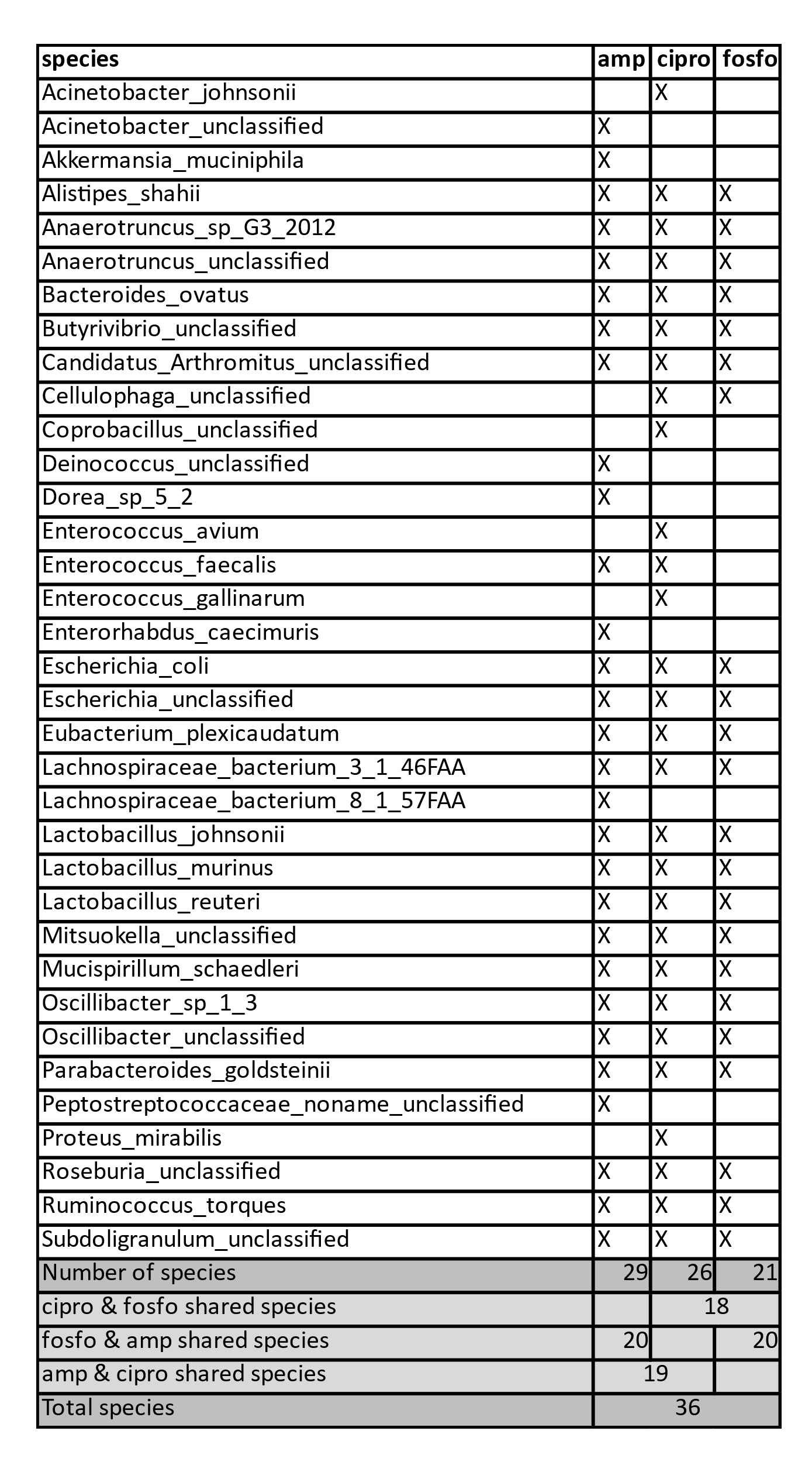
*

*Supplementary Table 7. Shared species*

*List o**f shared species across all three cohorts.*
